# Analysis of COVID-19–Related Croup and SARS-CoV-2 Variant Predominance in the US

**DOI:** 10.1001/jamanetworkopen.2022.20060

**Published:** 2022-07-01

**Authors:** Brian Lefchak, Amanda Nickel, Shea Lammers, Dave Watson, Gabrielle Z. Hester, Kelly R. Bergmann

**Affiliations:** 1 Department of Pediatric Emergency Medicine, Children’s Minnesota, Minneapolis; 2 Children’s Minnesota Research Institute, Children’s Minnesota, Minneapolis; 3 Department of Value and Clinical Excellence, Children’s Minnesota, Minneapolis

## Abstract

This cross-sectional study investigates the association of dominant SARS-CoV-2 variants with COVID-19–related croup.

## Introduction

Recent reports have found an association between SARS-CoV-2 and croup.^1,2,3^ We aimed to investigate whether SARS-CoV-2 variants were associated with the proportion of children with croup, as well as hospital and intensive care unit (ICU) admissions and racemic epinephrine (RE) treatment.

## Methods

This cross-sectional study was approved by the Children’s Minnesota Institutional Review Board with exemption of informed consent and adhered to the STROBE reporting guideline. The study included children aged 3 months to 8 years with *International Statistical Classification of Diseases and Related Health Problems, Tenth Revision* (*ICD-10*) diagnoses of COVID-19 and croup between January 1, 2021, and March 26, 2022, using data from 43 US children’s hospitals in the Pediatric Health Information System. We excluded children with croup-mimicking (eg, tracheitis) or complex chronic conditions.^4^ SARS-CoV-2 variant predominance (ie, Alpha or other variant, Delta, and Omicron) was determined from the Centers for Disease Control and Prevention (CDC) COVID Data Tracker and defined as more than 50% of SARS-CoV-2 diagnoses attributable to a particular variant. CDC data report estimated variant proportions based on genomic surveillance and account for longitudinal sampling between and within states. The primary outcome was encounters for COVID-19–related croup, summarized by week and variant predominance. Secondary outcomes included hospital and ICU admissions and RE treatment. We used mixed-effects logistic regression to evaluate the association of variant predominance with hospitalization and RE use after adjusting for age, sex, race and ethnicity, insurance, and census region. Analyses were conducted using Stata statistical software version 16 (StataCorp), and 2-sided *P* values < .05 were considered statistically significant.

## Results

We identified 5152 children with COVID-19–related croup (3329 [64.6%] boys; median [IQR] age, 17 [9-31] months) (Table). The proportion of children with COVID-19–related croup was significantly increased during Omicron (10.9%) compared with Alpha or other variant (4.1%) and Delta (3.6%) periods (*P* < .001) (Figure). Odds of hospitalization during Alpha or other variant (adjusted odds ratio [aOR], 1.28; 95% CI, 0.97-1.70) or Delta (aOR, 0.92; 95% CI, 0.74-1.15) periods were not significantly different compared with the period of Omicron predominance. Treatment with RE was less likely during the Delta period (aOR, 0.73; 95% CI, 0.61-0.87) and did not differ in the Alpha or other variant periods (aOR, 1.03; 95% CI, 0.81-1.31) compared with the period of Omicron predominance. The frequency of ICU admission was not statistically different across time periods (Table).

**Table.** Characteristics and Outcomes Associated With COVID-19–Related Croup Encounters by SARS-CoV-2 Variant

Characteristic	Children, No. (%)				*P* value
	Total (N = 5152)	Alpha or other variant (n = 345)	Delta (n = 806)	Omicron (n = 4001)	
Age, median (IQR), mo	17 (9-31)	19 (12-31)	23 (12-43)	16 (8-29)	<.001
Sex					
Boys	3329 (64.6)	234 (67.8)	539 (66.9)	2556 (63.9)	.31
Girls	1821 (35.4)	111 (32.2)	267 (33.1)	1443 (36.1)	
Race and ethnicity					
American Indian	18 (0.4)	1 (0.3)	0 (0.0)	17 (0.4)	<.001
Asian	235 (4.6)	10 (2.9)	30 (3.7)	195 (4.9)	
Hispanic or Latino	1495 (29.0)	93 (27.0)	189 (23.5)	1213 (30.3)	
Non-Hispanic Black	636 (12.3)	47 (13.6)	117 (14.5)	472 (11.8)	
Non-Hispanic White	2296 (44.6)	157 (45.5)	408 (50.6)	1731 (43.3)	
Pacific Islander	20 (0.4)	3 (0.9)	1 (0.1)	16 (0.4)	
Other^a^	452 (8.8)	34 (9.9)	61 (7.6)	357 (8.9)	
Insurance					
Government	2731 (53.0)	180 (52.2)	400 (49.6)	2151 (53.8)	<.001
Private	2135 (41.4)	149 (43.2)	365 (45.3)	1621 (40.5)	
Uninsured	202 (3.9)	5 (1.5)	29 (3.6)	168 (4.2)	
Other or unknown	84 (1.6)	11 (3.2)	12 (1.5)	61 (1.5)	
Census region					
South	1854 (36.0)	147 (42.6)	312 (38.7)	1395 (34.9)	<.001
Midwest	1639 (31.8)	112 (32.5)	303 (37.6)	1224 (30.6)	
West	1146 (22.2)	37 (10.7)	115 (14.3)	994 (24.8)	
Northeast	513 (10.0)	49 (14.2)	76 (9.4)	388 (9.7)	
Hospital admission	898 (17.4)	75 (21.7)	129 (16.0)	694 (17.4)	.06
LOS, median (IQR), d	1 (1-1)	1 (1-1)	1 (1-1)	1 (1-1)	.43
ICU admission	83 (1.6)	8 (2.3)	12 (1.5)	63 (1.6)	.55
Received RE	1750 (34.0)	127 (36.8)	228 (28.3)	1395 (34.9)	.001
RE doses, median (IQR)	2 (1-3)	2 (1-2)	2 (1-2)	2 (1-3)	.98
Dexamethasone	4691 (91.1)	312 (90.4)	722 (89.6)	3657 (91.4)	.23

Abbreviations: ICU, intensive care unit; LOS, length of stay; RE, racemic epinephrine.

^a^ Race and ethnicity were assessed to examine whether differences in SARS-CoV-2 variant predominance existed across populations. Race and ethnicity were determined by site-specific practices at each Pediatric Health Information System hospital, including self-report by a child’s guardian. Race was indicated as American Indian, Asian, Black, Pacific Islander, White, missing, multiracial, or other. Ethnicity was indicated as Hispanic or Latino or not Hispanic or Latino. In this study, race and ethnicity were categorized into 7 groups: American Indian, Asian, Hispanic or Latino, non-Hispanic Black, non-Hispanic White, Pacific Islander, and other (includes missing, multiracial, and race indicated as “other”).

**Figure.**
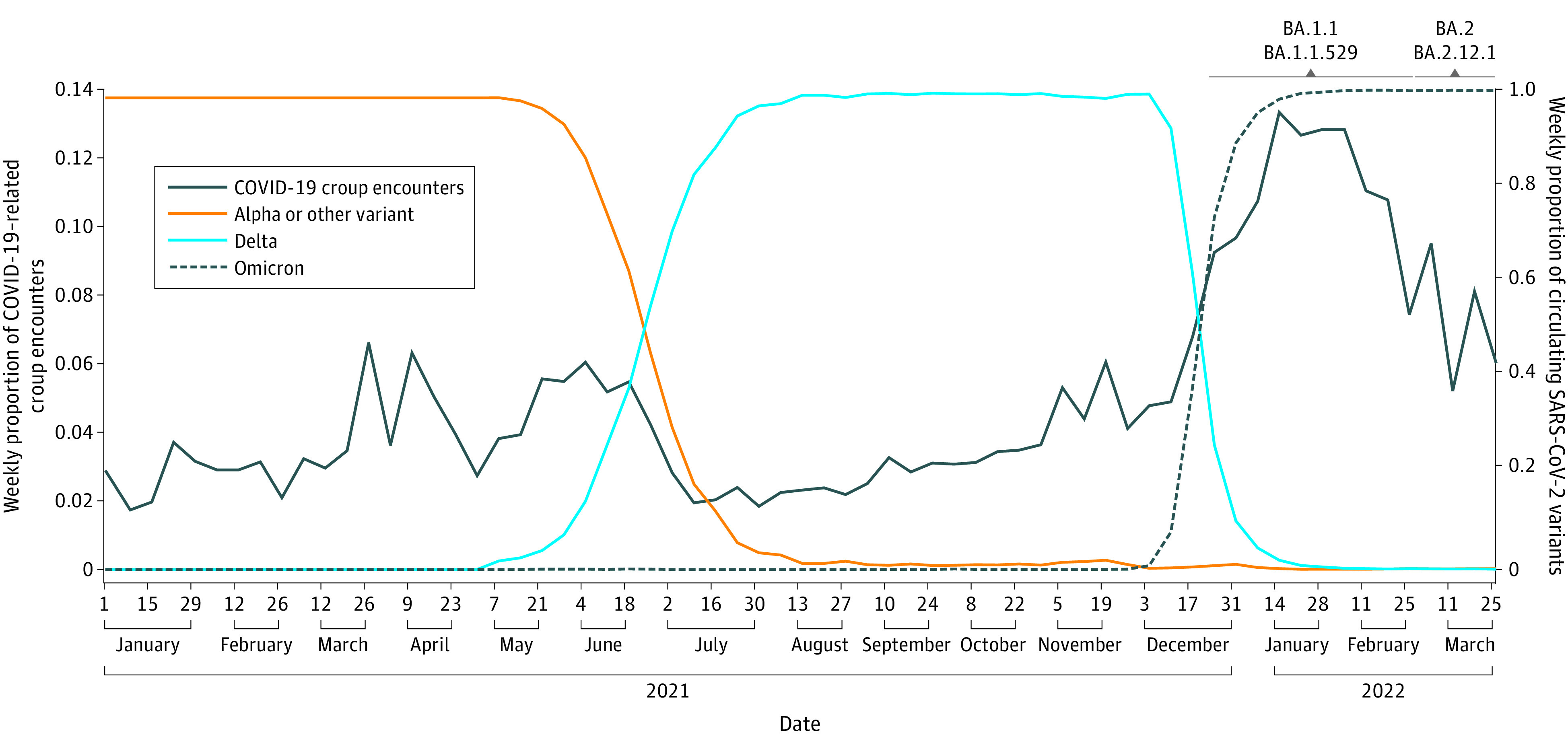
Proportions of Weekly COVID-19–Related Croup Encounters and SARS-CoV-2 Variants COVID-19–related croup encounters were identified using Pediatric Health Information System data, and SARS-CoV-2 variant data was obtained from the Centers for Disease Control and Prevention COVID Data Tracker. Alpha or other includes Alpha (B.1.1.7), Beta (B.1.351), Epsilon (B.1.427 and B.1.429), Eta (B.1.525), Gamma (P.1), Iota (B.1.526), and Kappa (B.1.617.1) variants. Delta and Omicron include Delta (B.1.617.2) and Omicron (B.1.1.529, BA.1.1, BA.2, and BA.2.12.1) variants.

## Discussion

The results of this cross-sectional study expand on recent single-center studies^1,2^ showing that hospitalizations for COVID-19–related croup increased after the onset of the Omicron variant. A more recent national investigation^3^ found that the percentage of children diagnosed with SARS-CoV-2 hospitalized with upper-airway infections increased significantly from pre-Omicron (1.4%) compared with Omicron (4.1%) periods. The hospitalization rate was higher in our study, which may be associated with use of *ICD-10* codes rather than positive SARS-CoV-2 test results for COVID-19 data.

Findings for association with COVID-19–related croup severity were mixed in our study. We noted a significant increase in the proportion of children requiring RE during Alpha or other variant and Omicron periods compared with the period of Delta predominance. However, we also observed no difference in the median number of RE doses, which was comparable to an estimate prior to COVID-19.^5^ The overall ICU admission rate in our sample was lower than a rate described prior to COVID-19,^5^ which may be associated with constraints on ICU capacity or, alternatively, less severe illness.

Our study has several limitations. Hospital practice changes, including limitation of aerosol-generating procedures early in the pandemic, may have influenced RE administration. It is also possible that conditions other than croup influenced hospitalization rates.

Given that COVID-19 is likely to become endemic, our findings suggest that pediatric health systems should consider variation in SARS-CoV-2 phenotypes and their association with patient care. This may be especially true when other viral infections lead to surges in patient volume.
